# A persistent gallstone—an unusual cause of colonic obstruction in sigmoid adenocarcinoma

**DOI:** 10.1093/jscr/rjag320

**Published:** 2026-04-28

**Authors:** Benedict Blacket, Kasmira Wilson, Thomas Barnes

**Affiliations:** University of Oxford, Department of Physiology, Anatomy and Genetics, Sherrington Rd, Oxford, OX1 3PT, United Kingdom; University of Oxford Hospitals NHS Foundation Trust, Headley Way, Headington, Oxford OX3 9DU, United Kingdom; University of Oxford Hospitals NHS Foundation Trust, Headley Way, Headington, Oxford OX3 9DU, United Kingdom; University of Oxford Hospitals NHS Foundation Trust, Headley Way, Headington, Oxford OX3 9DU, United Kingdom

**Keywords:** gallstone ileus, bowel obstruction, adenocarcinoma, malignancy

## Abstract

An 88-year-old woman presented with acute post-prandial abdominal pain, nausea, and rigors, with biochemical evidence of obstructive jaundice. Computed tomography demonstrated choledocholithiasis with acute cholecystitis and an incidental segment of thickened descending colon. Following endoscopic sphincterotomy with stone extraction and subsequent laparoscopic cholecystectomy, she was discharged home. She re-presented 6 days later with constipation and lower abdominal pain. Repeat imaging revealed large bowel obstruction with marked cecal dilatation due to migration of a gallstone impacted at the site of the previously identified colonic narrowing. Emergency Hartmann’s procedure was performed. Histopathology confirmed a moderately differentiated adenocarcinoma of the colon (pT4aN0R0). This case highlights a rare complication of gallstone migration resulting in colonic obstruction, which likely expedited the diagnosis and definitive management of underlying colorectal malignancy.

## Introduction

Gallstone disease is common, with prevalence increasing with age, obesity, and metabolic syndrome [1]. Most individuals remain asymptomatic however, ~10% of patients will develop complications, including cholecystitis, choledocholithiasis, pancreatitis, and cholangitis [2]. Gallstone ileus is a rare but serious complication, occurring when a gallstone migrates into the gastrointestinal tract and causes mechanical bowel obstruction, typically following formation of a cholecystoenteric fistula [3].

Gallstone ileus accounts for ~1%–4% of all cases of bowel obstruction, with most stones impacting within the small intestine, particularly at the terminal ileum [4, 5]. Large bowel obstruction due to gallstone ileus is uncommon, representing only 4%–8% of reported gallstone ileus cases, and is usually associated with underlying colonic pathology that results in luminal narrowing, such as diverticular disease, inflammatory strictures, or malignancy [6].

Endoscopic retrograde cholangiopancreatography (ERCP) with sphincterotomy is the gold standard management for choledocholithiasis and typically permits safe passage of gallstones into the bowel lumen [1]. While these stones are usually small and pass without consequence, gallstone ileus following ERCP has been described in the presence of co-existing bowel pathology [7].

We report a rare case of gallstone ileus causing large bowel obstruction at the site of an occult sigmoid adenocarcinoma following ERCP, which expedited diagnosis and definitive management of the underlying colorectal malignancy.

## Case report

**Figure 1:**
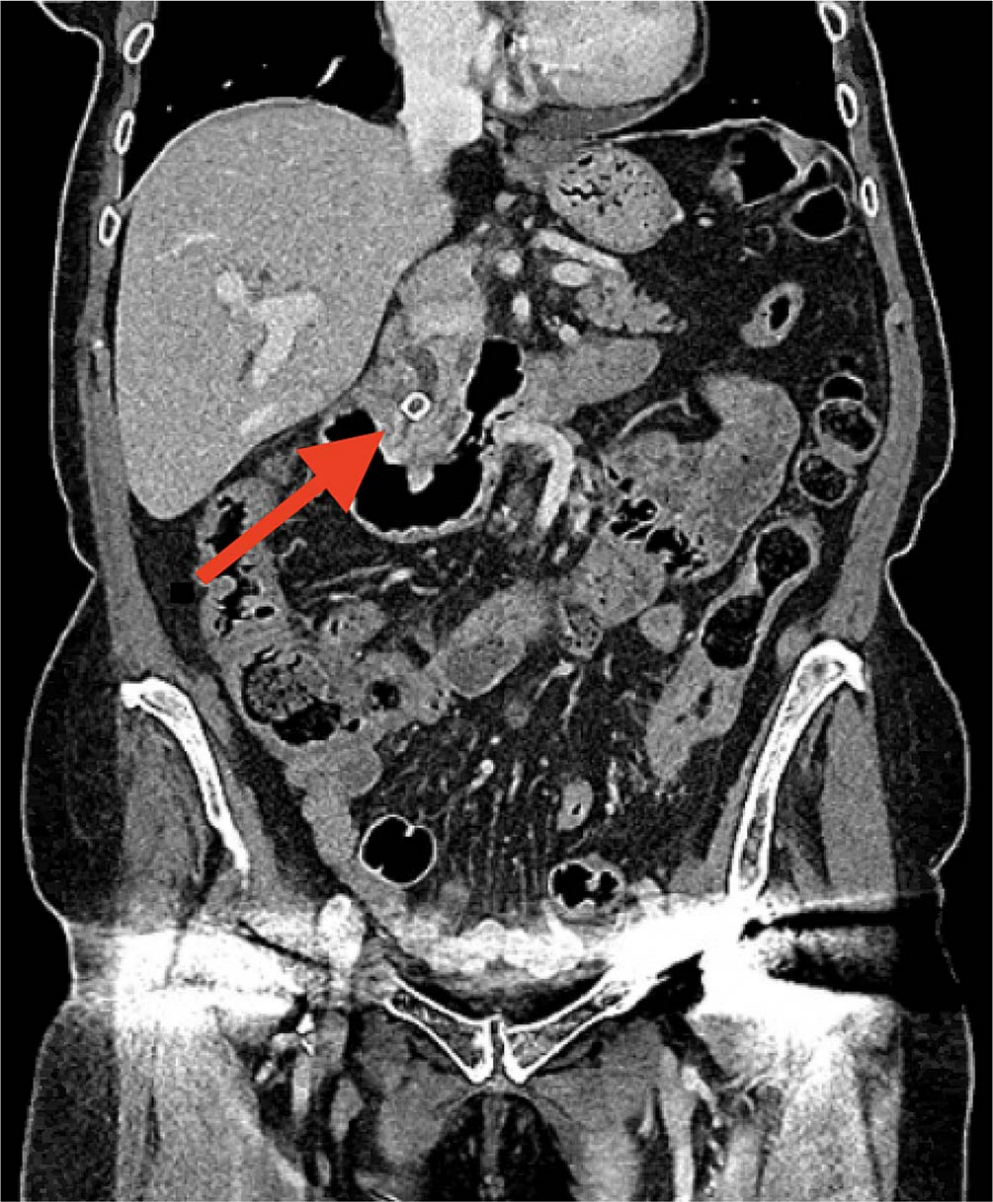
Initial CT demonstrating the obstructing distal common bile duct stone. *This image was obtained at University of Oxford Hospitals NHS Foundation Trust with patient consent.*

An 88-year-old female presented with a 24-h history of generalized post-prandial abdominal pain, nausea, and rigors. She had a past medical history of asthma, hypertension, and gastroesophageal reflux disease and is independent with an Eastern Cooperative Oncology Group score of 1. Serum biochemistry demonstrated cholestatic liver derangement and bilirubinaemia of 80 umol/l. An initial computed tomography (CT) was performed (Fig. 1) which demonstrated a 10 mm obstructive gallstone within the distal common bile duct, and cholecystitis. Additionally, the scan demonstrated a thickened segment of descending colon which prompted referral for outpatient colonoscopy. She was commenced on intravenous antibiotics and referred for ERCP where a sphincterotomy was performed to remove the bile duct stone. Five days following this procedure, she underwent an uncomplicated laparoscopic cholecystectomy and was discharged home.

**Figure 2:**
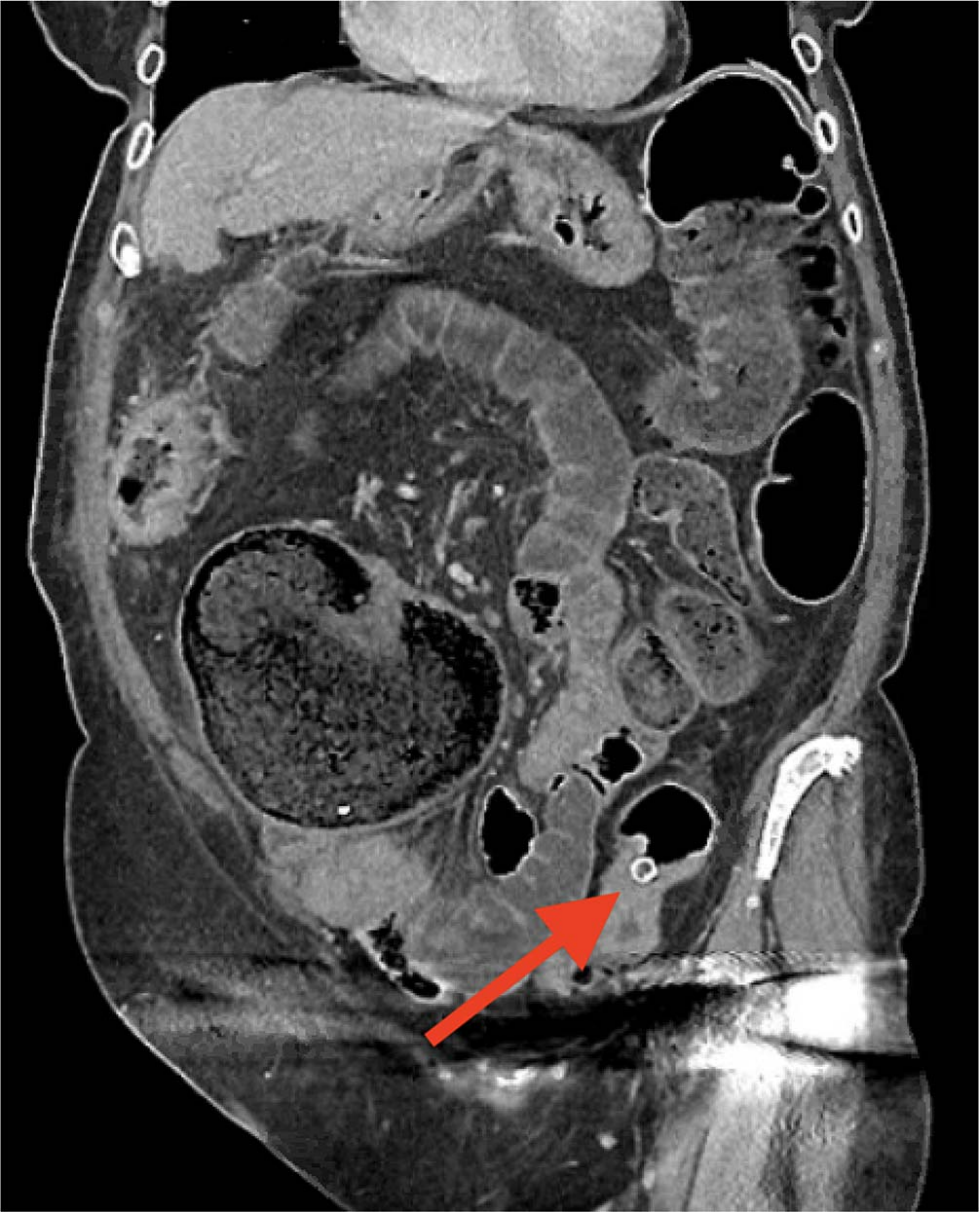
Subsequent CT demonstrating large bowel obstruction and migrated gallstone lodged at the distal descending colon. *This image was obtained at University of Oxford Hospital NHS Foundation Trust with patient consent.*

Six days later, she re-presented to our unit with generalized lower abdominal pain with 7 days of constipation, having not opened her bowels following the cholecystectomy. A second CT scan was performed (Fig. 2) which demonstrated a large bowel obstruction with gross cecal dilatation secondary to a migrated gallstone which had become lodged at the narrowing in the thickened distal descending colon. She subsequently underwent an emergency open Hartmann’s procedure the following day. Histopathology demonstrated a moderately differentiated adenocarcinoma with clear margins and no lymph node involvement (pT4a, N0, R0). There was no evidence of pulmonary metastatic disease on a CT Thorax, and a completion colonoscopy was negative. She met with the oncology team and on the balance of risks and benefits, she has opted for surveillance rather than systemic chemotherapy.

## Discussion

Colonic gallstone ileus is a rare but clinically significant cause of bowel obstruction, accounting for 1% of cases and predominantly affecting elderly patients with existing comorbidities [6]. In this case, the persistent obstructive nature of a migrated gallstone resulted in acute large bowel obstruction which likely hastened the diagnosis and definitive treatment of an occult colorectal malignancy. This unusual sequence of events highlights a rare interaction between biliary intervention and colonic pathology.

Unlike classical gallstone ileus, which most commonly results in small bowel obstruction, large bowel involvement is uncommon and typically occurs in the presence of pre-existing colonic pathology causing luminal narrowing [6–8]. In contrast to the classical mechanism involving cholecystoenteric fistulation, gallstone migration following ERCP and sphincterotomy represents an alternative pathway by which gallstones may enter the gastrointestinal tract.

To our knowledge, only one previous case has reported on a coinciding presentation of gallstone ileus and colorectal adenocarcinoma resulting in bowel obstruction [9]. Marie *et al.* described a patient with gallstone ileus and a partially obstructing colonic malignancy, highlighting the diagnostic and therapeutic challenges posed by the coexistence of two obstructive pathologies affecting different bowel segments [9]. In contrast to our case, the obstruction in this report resulted from cholecystoenteric fistulation and caused small bowel obstruction in the presence of a coinciding sigmoid mass, without direct gallstone impaction at the site of the colonic malignancy. In our case, gallstone migration occurred following ERCP and sphincterotomy, resulting in direct impaction at the site of an occult sigmoid adenocarcinoma. These parallel findings emphasize the importance of maintaining vigilance for underlying colonic pathology when gallstone ileus is identified, particularly in elderly patients.

There is no consensus on the management of gallstone ileus with clinicians often considering individual patient physiology, stone location, and underlying pathology. Reported strategies include enterolithotomy, segmental colectomy, or endoscopic extraction [3]. More broadly, management of gallstone ileus traditionally involves surgical removal of the obstructing stone, with the extent of bowel resection dictated by tissue viability, stone location, and associated pathology [6, 10]. In our case, where malignancy was suspected, an emergency oncological resection was indicated as per guidelines for malignant acute left-sided colonic obstruction [11]. Emergency Hartmann’s procedure provided definitive relief of obstruction while achieving curative resection of a pT4aN0 sigmoid adenocarcinoma.

This case demonstrates how an acute surgical complication may paradoxically expedite oncological diagnosis and management. The migrated gallstone precipitated bowel obstruction, prompting urgent surgical intervention, and enabling early identification and complete resection of colorectal cancer. Clinicians should therefore maintain an index of suspicion for underlying colorectal pathology in patients presenting with gallstone ileus, particularly following biliary intervention, such as ERCP, and incidental colonic abnormalities identified on imaging should prompt timely investigation.
